# Supplementary material for: Specialized shuttle proteins recognize Type IX secretion signals and target effectors to their final destinations in Flavobacterium johnsoniae
Source: Commun Biol. 2025 Nov 14;8:1566. doi: 10.1038/s42003-025-08926-8 (PMC12618676; doi:10.1038/s42003-025-08926-8)
Supplement: Supplementary file 1 — Supplementary Information [file 42003_2025_8926_MOESM1_ESM.pdf]

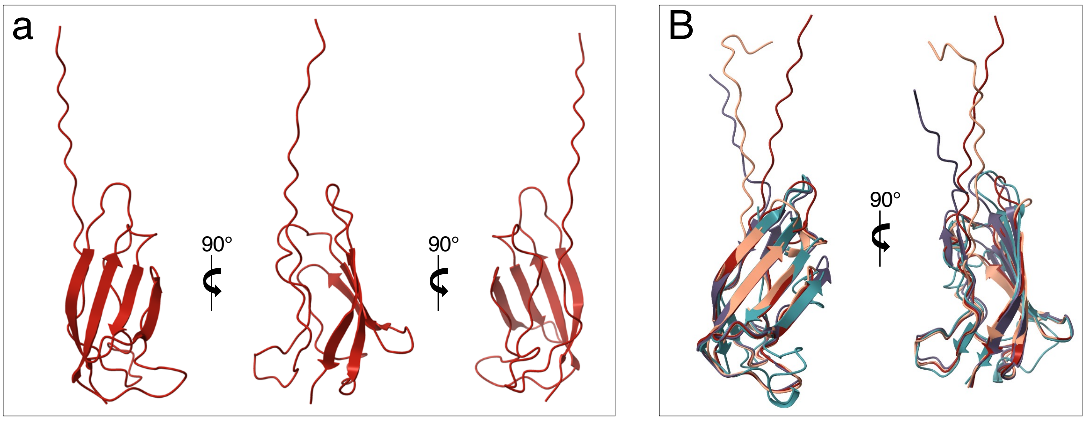

C

Structure pair	Sequence alignment score	Number of aligned residues	rmsd of pruned atom pairs (Å)
SprB/Fjoh_1123	238.8	77	0.624
SprB/Fjoh_3952	131.4	67	0.976
SprB/Fjoh_4750	161.7	72	0.665

Supplementary Figure 1. Predicted structure of the CTD of SprB, and its alignment with CTDs of Fjoh_1123 (orange), Fjoh_3952 (blue) and Fjoh_4750 (violet). (A) The structure of the last 100 amino acids of SprB was predicted using AlphaFold3 (PTM score of 0.82) (Abramson et al., 2024) and is shown in three orientations. **(B)** The CTDs of Fjoh_1123, Fjoh_3952 and Fjoh_4750 have been predicted using AlphaFold3 (Abramson et al., 2024), with respective pTM scores of 0.83, 0.85 and 0.86. These predicted structures were superposed to the predicted structure of the CTD of SprB using ChimeraX-1.9. **(C)** Sequence alignment scores, number of aligned residues and rmsd of the predicted structure of SprB CTD aligned to the predicted structure of the CTDs of Fjoh_1123, Fjoh_3952 or Fjoh_4750.

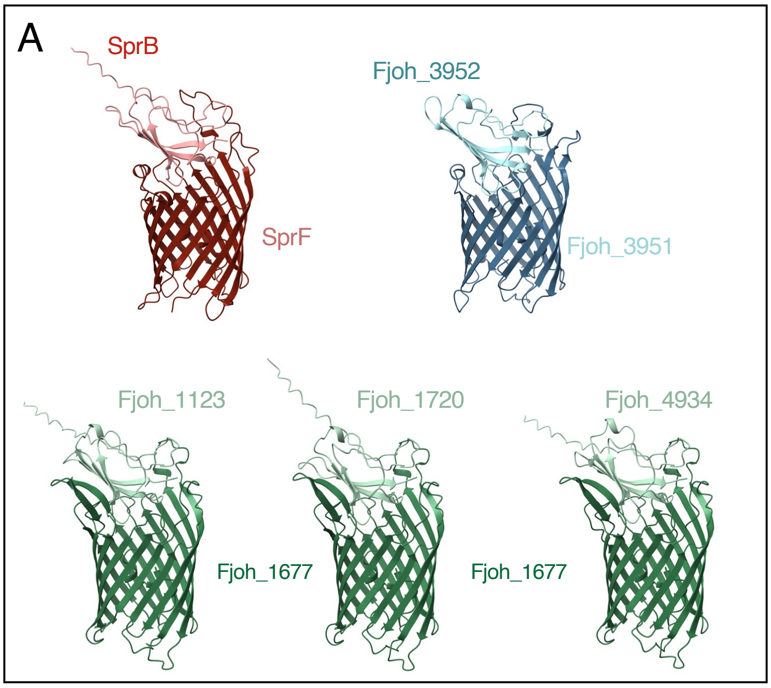

B

CTD/shuttle pair	ipTM score
SprB/SprF	0.8
3952/3951	0.88
1123/1677	0.86
1720/1677	0.85
4934/1677	0.87

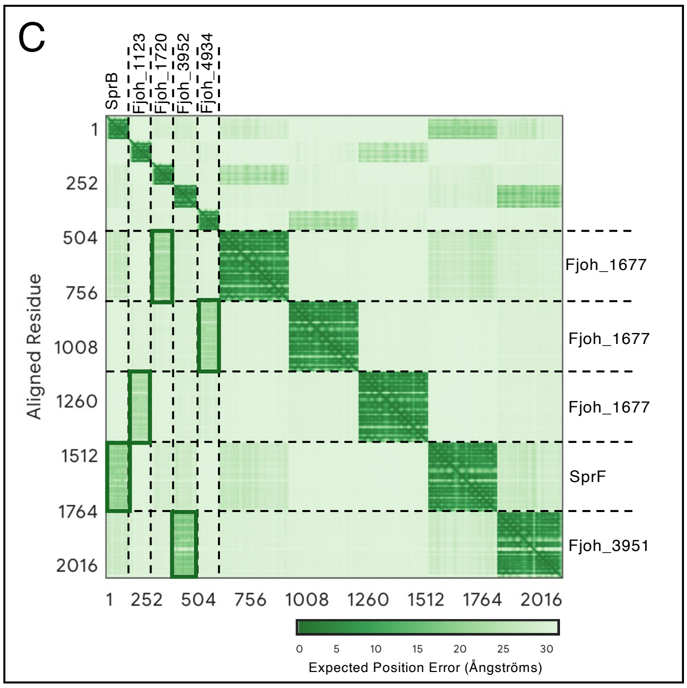

Supplementary Figure 2. AlphaFold3 model predictions of specific CTD/shuttle pairs. (A) CTD/shuttle complexes were predicted using the last 100 amino acids of SprB (red shades), Fjoh_3952 (blue shades), Fjoh_1123, Fjoh_1720, and Fjoh_4934 (green shades), along with the corresponding shuttle sequences lacking their N-terminal signal peptides. **(B)** For each CTD/shuttle protein pair prediction, the ipTM score of the AlphaFold3 prediction is presented in the Table. **(C)** Predicted aligned error plot of these five CTDs and shuttles complexes, predicted at the same time, showed that each CTD was associated with its cognate shuttle. Dashed black lines delineate boundaries between tested proteins. Green intensity indicates the strength of contact frequency between amino acid residues. Thus, intra-molecular (diagonal) and inter-molecular (off-diagonal, green boxes) regions of interactions are dark green (high confidence).

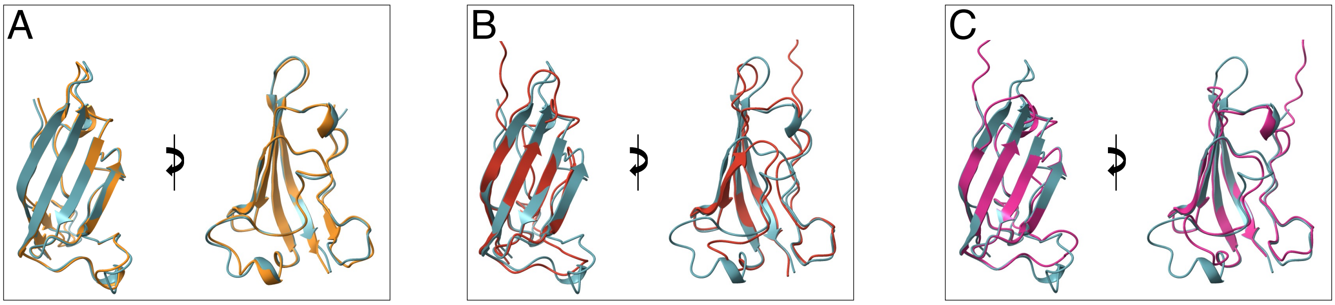

D

Structure pair with Fjoh_3952	Sequence alignment score	Number of aligned residues (/100)	rmsd of pruned atom pairs (Å)
Fjoh_1645	405.9	99	0.608
SprB	131.4	67	0.976
Fjoh_3971	221.6	72	0.621

Supplementary Figure 3. Predicted structural alignments of the CTD of Fjoh_3952 with the CTD of Fjoh_1645, SprB or Fjoh_3971. The structure of the last 100 amino acids of Fjoh_3952 (blue), Fjoh_1645 (orange), SprB (red), and Fjoh_3971 (pink) have been predicted using AlphaFold3 (Abramson et al., 2024), with pTM scores of 0.85, 0.84, 0.82 and 0.8, respectively. Predicted structures of the CTDs of Fjoh_1645 **(A)**, SprB **(B)** or Fjoh_3971 **(C)** were aligned to the predicted structure of the CTD of Fjoh_3952 using ChimeraX-1.9. **(D)** Sequence alignment scores, number of aligned residues and rmsd of the predicted structure of Fjoh_3952 CTD aligned to predicted structures of the CTDs of SprB, Fjoh_1645 and Fjoh_3971.

A

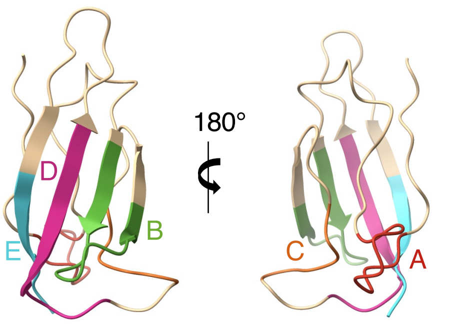

B

A B C D E

$SprB$:
CTDTKDYYIEYVDVCLDNYFTPNGDGVNDTWGPGCTNIYNHLKFSIFDRYGRVIAKYTYGQKWDGRYNGEELPSGDYWYVLKLNDENDGREFVGHFTLYR

$SprB_{motA}$:
CTDTKDYYIEYVDVCLDNYFTPLGLGVALTWGPGCTNIYNHLKFSIFDRYGRVIAKYTYGQKWDGRYNGEELPSGDYWYVLKLNDENDGREFVGHFTLYR

$SprB_{motB}$:
CTDTKDYYIEYVDVCLDNYFTPNGDGVNDTWGPGCTNIYNHLKFAIMDMMGMVIAKYTYGQKWDGRYNGEELPSGDYWYVLKLNDENDGREFVGHFTLYR

$SprB_{motC}$:
CTDTKDYYIEYVDVCLDNYFTPNGDGVNDTWGPGCTNIYNHLKFSIFDRYGRVIAKYTYGQKWLGMYLGEELPSGDYWYVLKLNDENDGREFVGHFTLYR

$SprB_{motD}$:
CTDTKDYYIEYVDVCLDNYFTPNGDGVNDTWGPGCTNIYNHLKFSIFDRYGRVIAKYTYGQKWDGRYNGEELPSGLYMYGLKLNDENDGREFVGHFTLYR

$SprB_{motE}$:
CTDTKDYYIEYVDVCLDNYFTPNGDGVNDTWGPGCTNIYNHLKFSIFDRYGRVIAKYTYGQKWDGRYNGEELPSGDYWYVLKLNDENDGREFVGMFVLMR

$SprB_{swap}$:
CTDTKDYYIEYVDVCLDNYISLNGDNMNETWGPGCTNIYNHLKVQIFNRWGVLVFKYTYGQKFKGFSEGEELPEGTYYYIVKLNDENDGREFVGYLYLIK

C

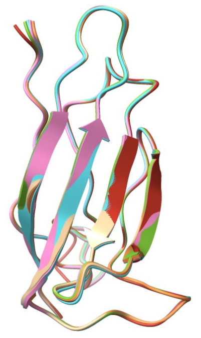

D

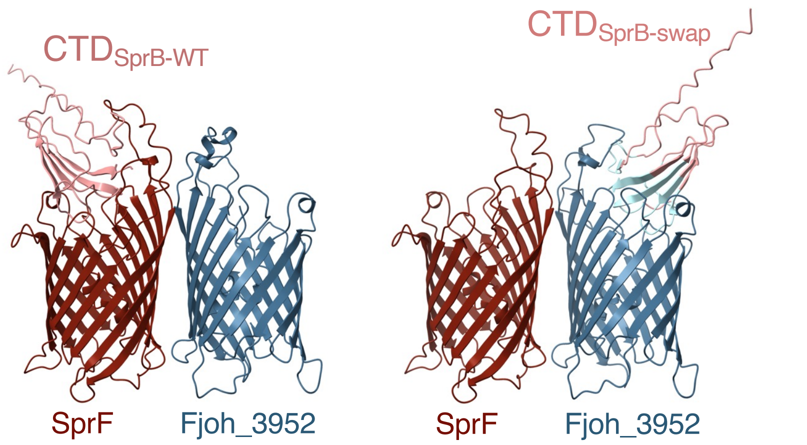

Supplementary Figure 4. Structure prediction suggests that the five conserved motifs in type B CTDs are required for the specific recognition of the CTD by its cognate shuttle. (A) The five Type B CTDs conserved motifs have been colored in red, green, orange, pink and light blue respectively on the AlphaFold3 model of SprB CTD. **(B)** Mutations and swap of the conserved motifs in the CTD of SprB. The same colors as in **(A)** were used to highlight the conserved motifs in the sequence of the CTD. The sequences of SprB CTD with individual motifs mutated or with the 5 motifs from Fjoh_3952 are presented. Mutation in the motifs are shown in bold black and Fjoh_3952 motifs are colored in teal blue. **(C)** Structural alignment of the WT and mutated versions of SprB CTD. The predicted structure of the WT CTD is shown in beige, while each motif mutant is colored according to the scheme used in panel **A**. **(D)** AlphaFold3 was used to assay the interaction of SprB CTD (light red) with SprF (dark red) or Fjoh_3951 (dark blue), with the wild-type CTD sequence ($CTD_{SprB\text{-}WT}$, *left panel*) or while swapping SprB motifs with the conserved motifs of Fjoh_3952 ($CTD_{SprB\text{-}swap}$, *right panel*). The results suggest that the five motifs of Fjoh_3952 are necessary and sufficient for its specific recognition by Fjoh_3951.

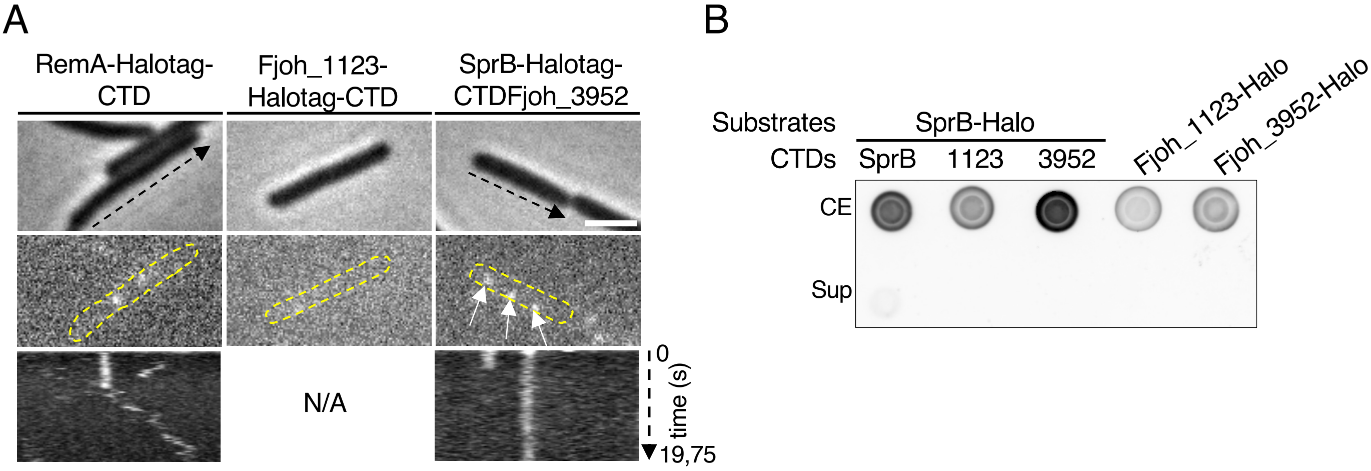

Supplementary Figure 5. Localization and behaviour of Fjoh_1123-HaloTag-CTD, RemA-HaloTag-CTD and SprB-HaloTag-CTD$_{\mathbf{Fjoh_3952}}$ fusions. **(A)** For all strains, cells were sandwiched between an agarose pad (2%) and a glass coverslip to significantly limit cell movement to facilitate fluorescence signal acquisition and analysis. Fluorescence was recorded at 250 ms intervals for several seconds. The phase contrast image (top panel), the first frame (middle panel), and the kymograph of the fluorescence signal (bottom panel) are shown when applicable. A representative cell is shown. Scale bar, 2 µm. **(B)** Secretion profile of the HaloTag fusions. The secretion of the different HaloTag fusions used in the study was monitored. Total cell extract (CE) and supernatant (Sup) were separated and analyzed by anti-HaloTag immunoblotting. Uncropped blots are shown in Supplementary Fig. 8.

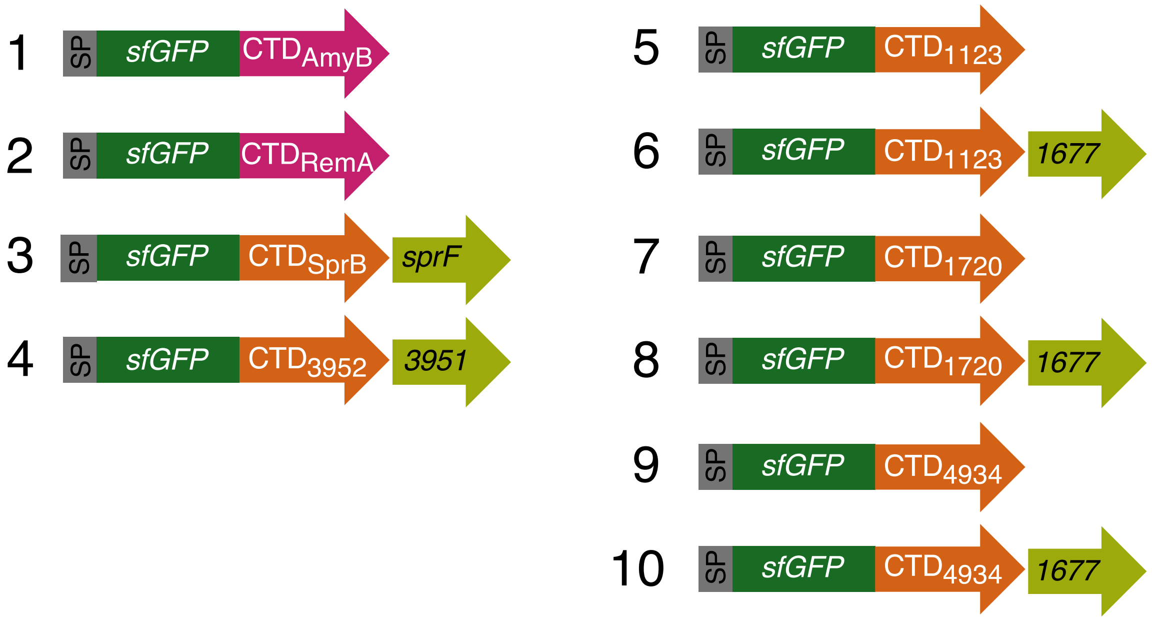

Supplementary Figure 6. Schematics of the fusions used in secretion and co-immunoprecipitation assays. For constructs 1, 2, 5, 7 and 9, the plasmid is designed to produce (expression from the *remA* promoter) a fusion constituted of the signal peptide region of the RemA adhesin (SP), sfGFP, a linker, and the desired CTD region. For constructs 3, 4, 6, 8 and 10, the plasmid is designed to express (from the *remA* promoter as an operon) the desired sfGFP-CTD fusion and the Fjoh_1677 shuttle protein.

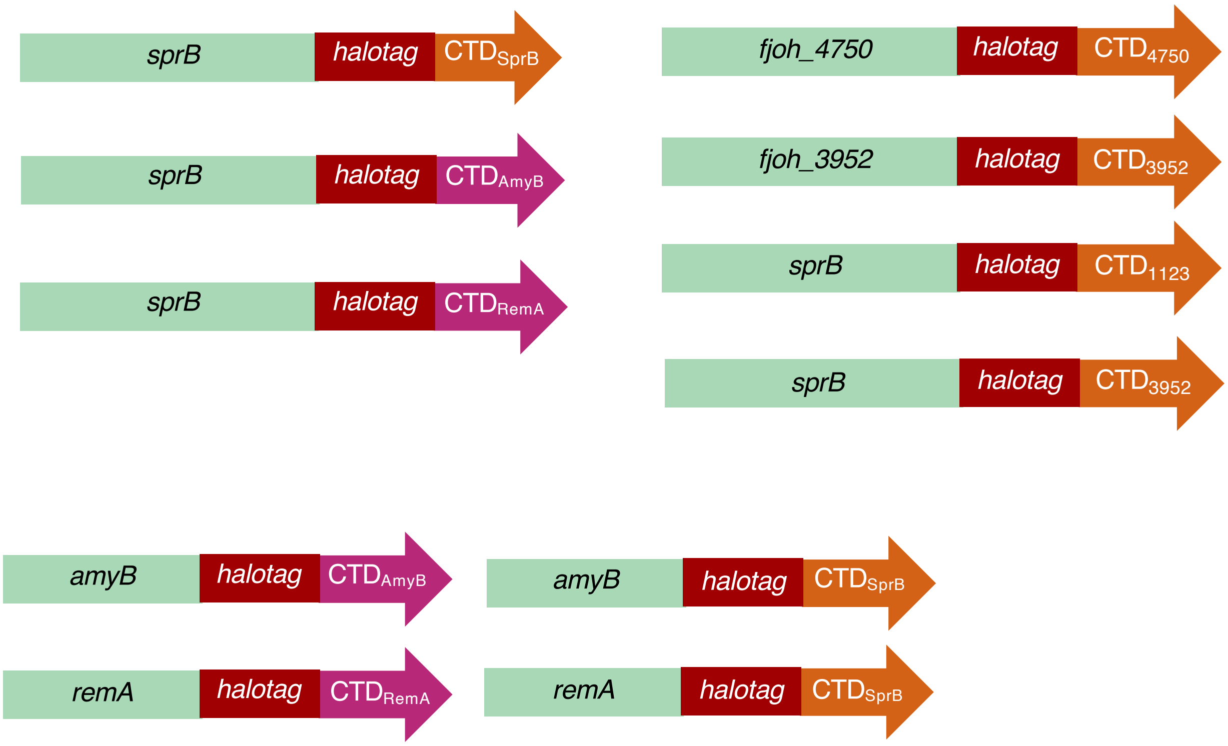

Supplementary Figure 7. Schematics of the Halotag fusions observed by fluorescence microscopy. All constructs show the same structure. HaloTag is inserted upstream of the CTD region, which is either the native CTD or the CTD or another Type A (pink) or Type B substrate (brown).

Supplementary Figure 8

(Fig. 1B)

Chemiluminescence image

Epi-illumination image

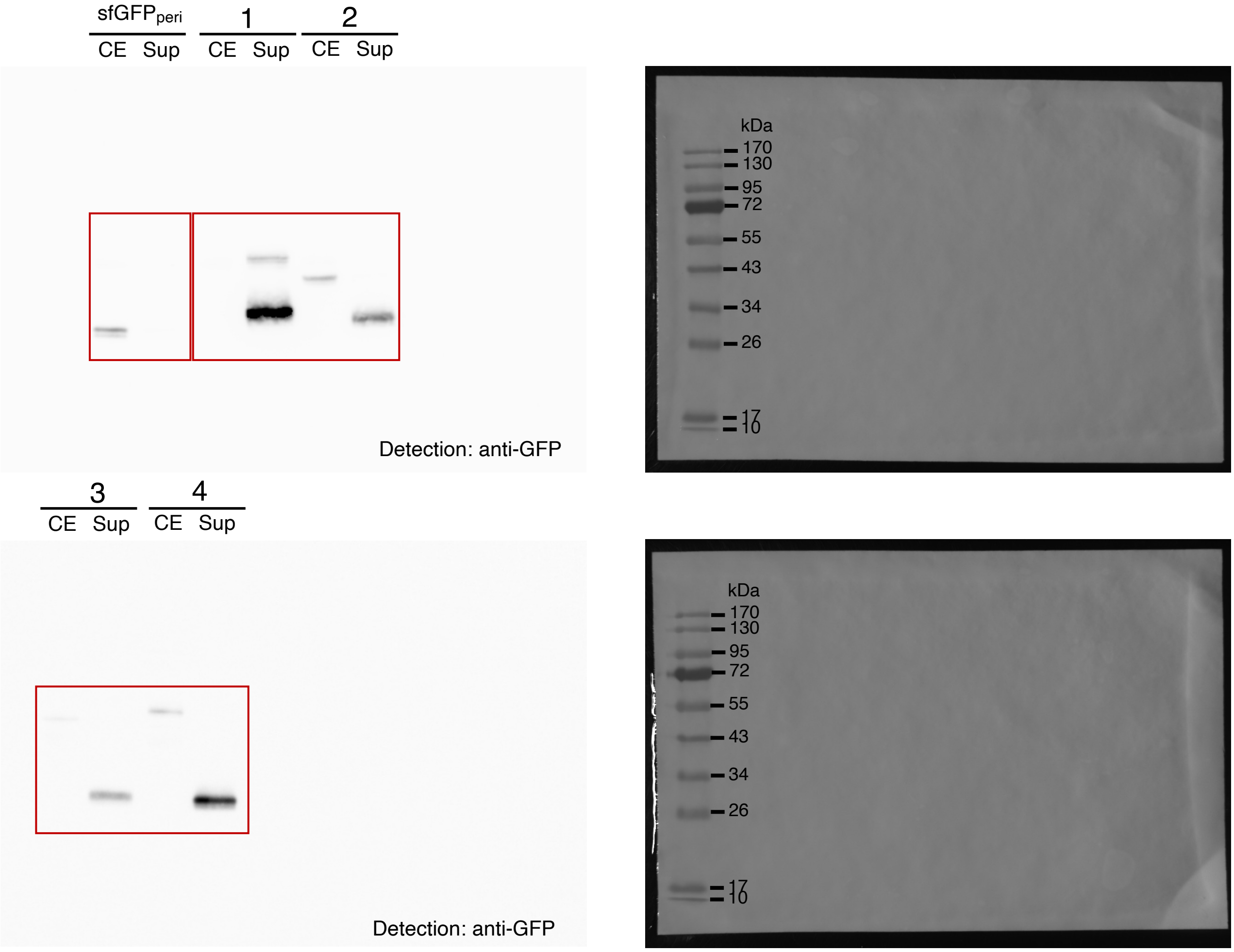

(Fig. 1C)

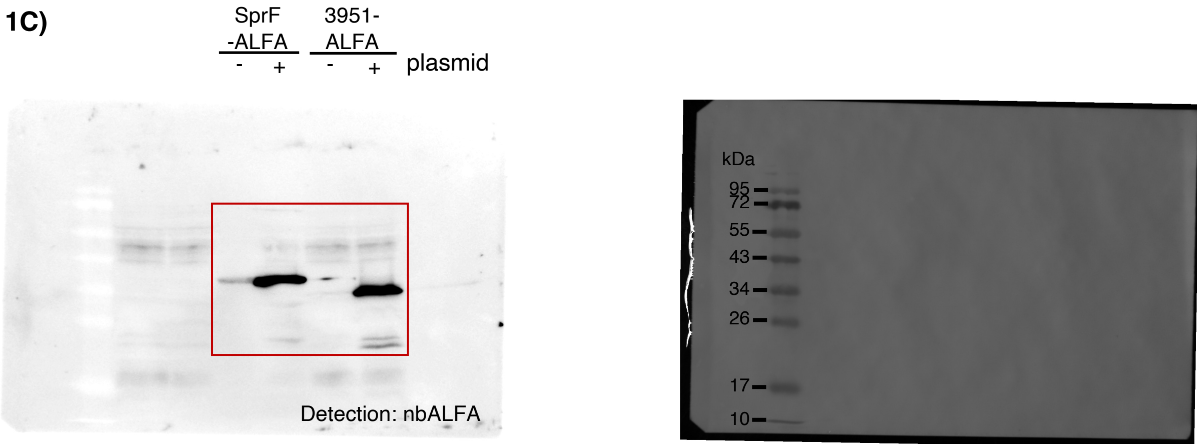

(Fig. 1D)

Chemiluminescence image

Epi-illumination image

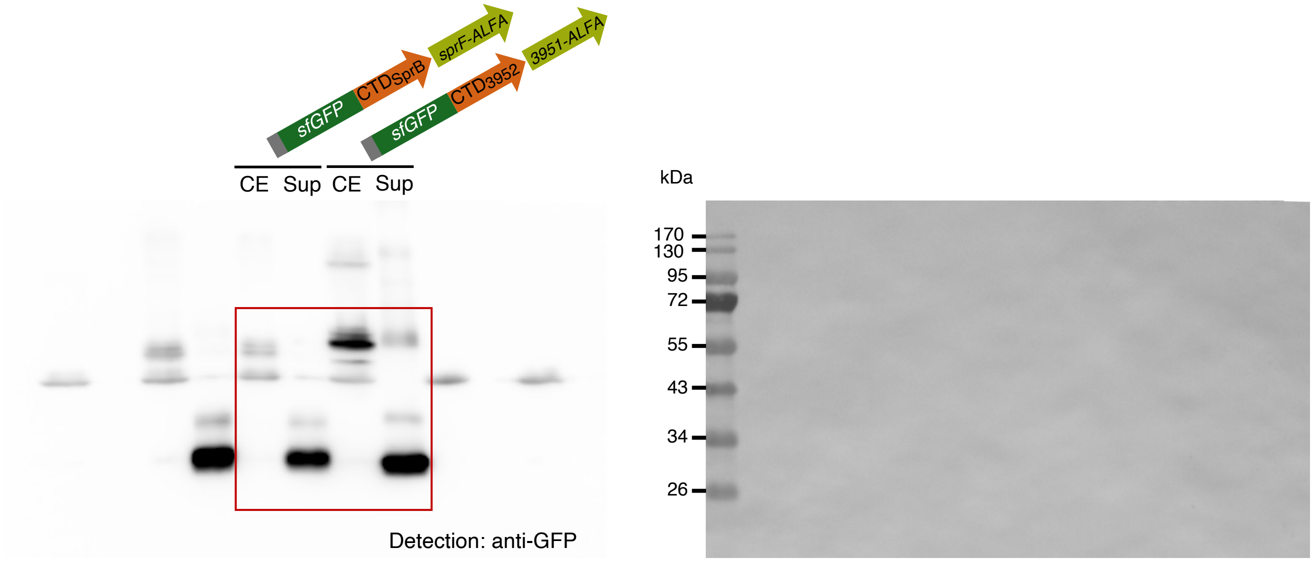

(Fig. 3B)

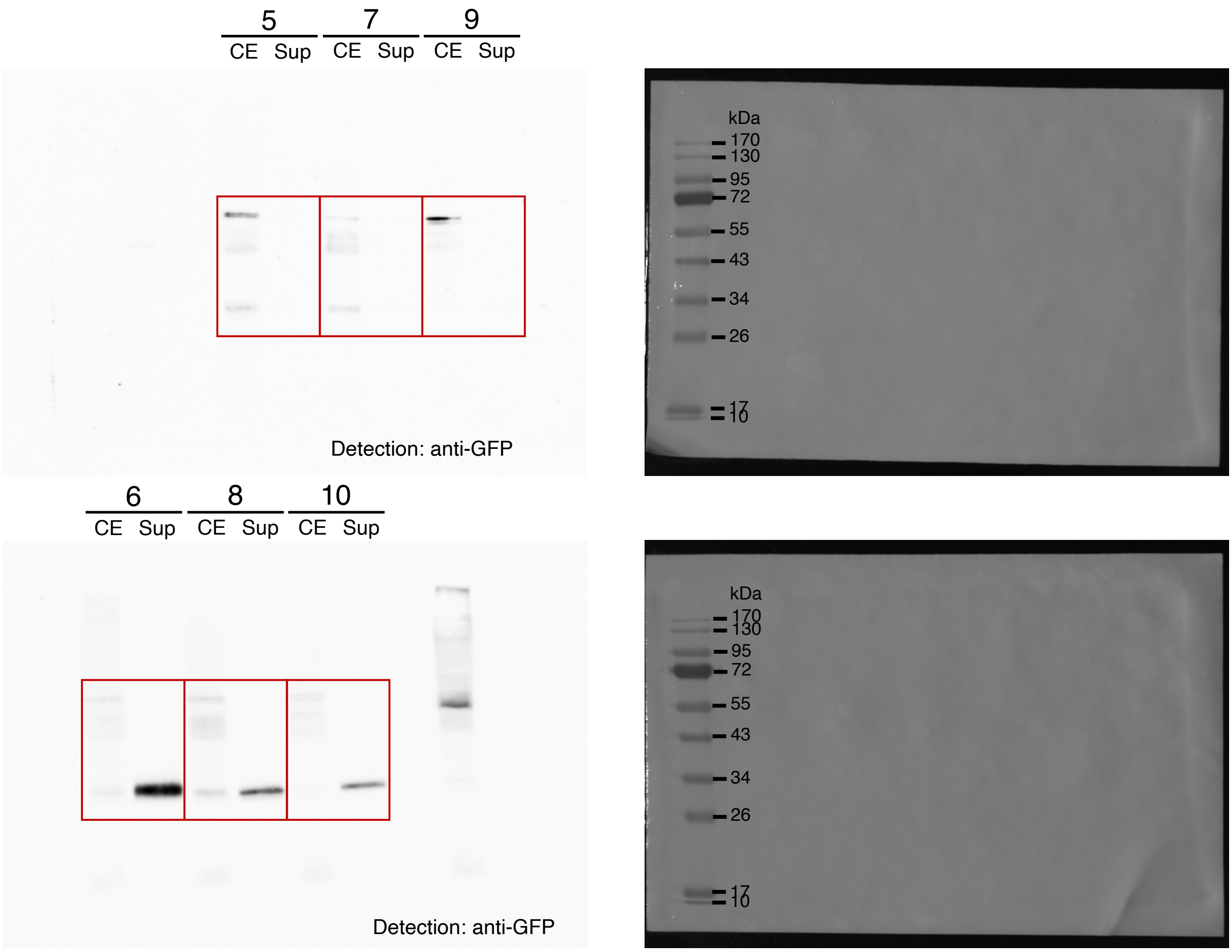

(Fig. 4)

Chemiluminescence image

Epi-illumination image

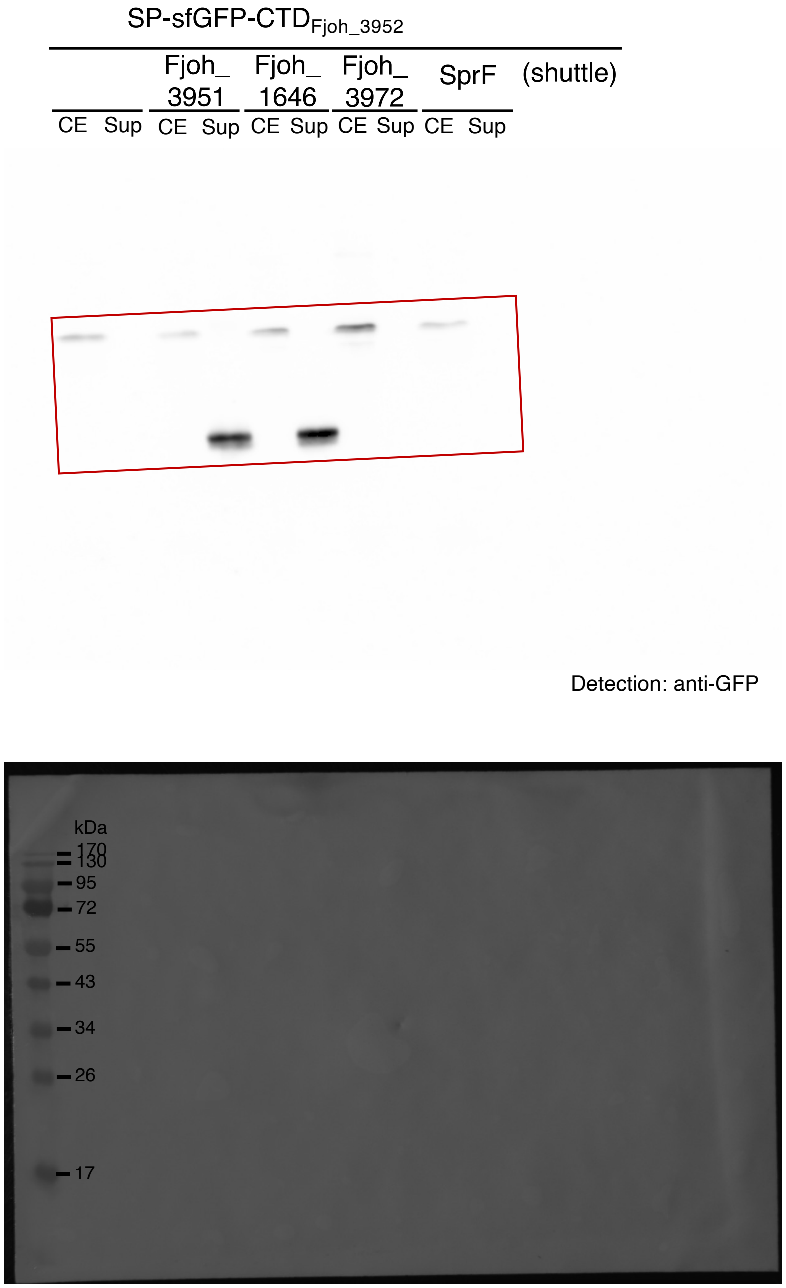

(Fig. 5B)

Chemiluminescence image

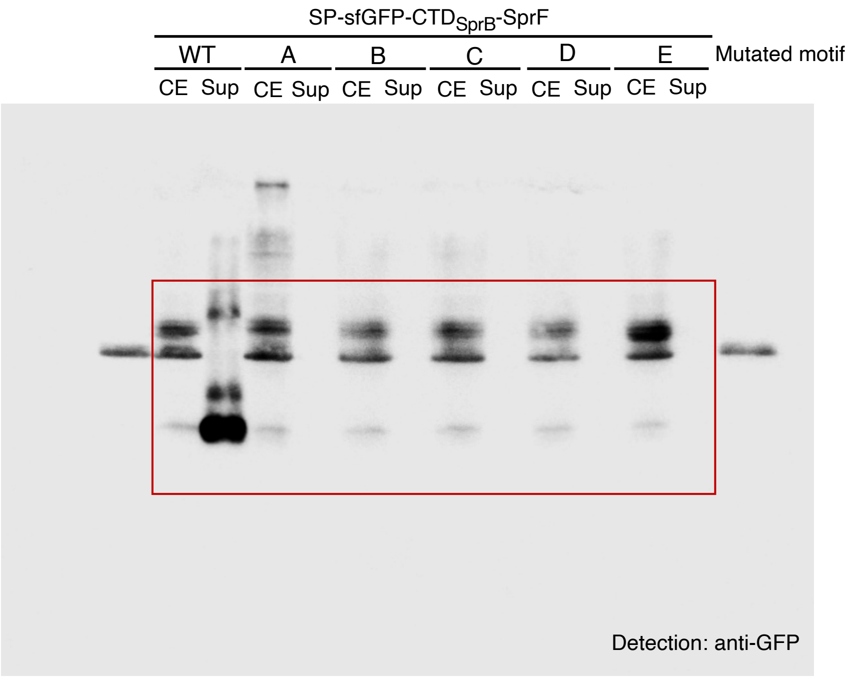

Epi-illumination image

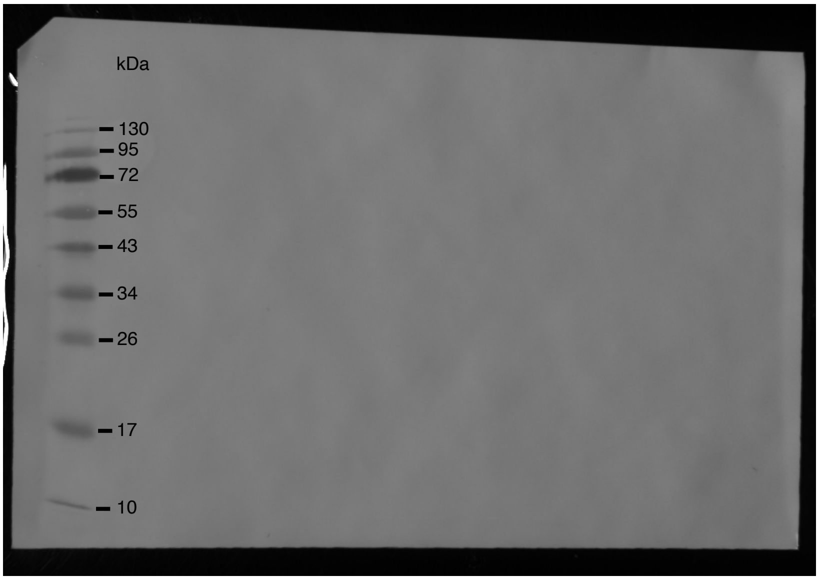

(Fig. 5C)

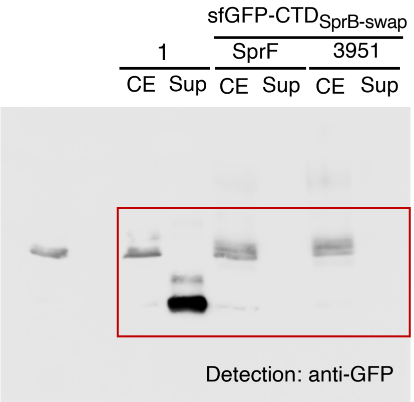

Chemiluminescence image

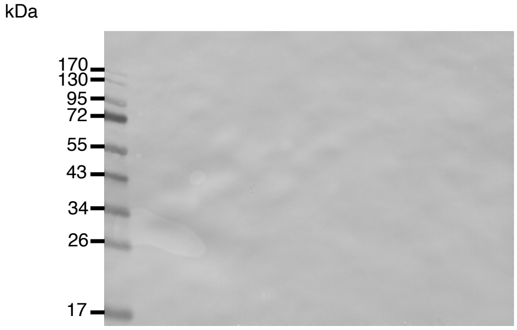

Epi-illumination image

(Fig. 6E)

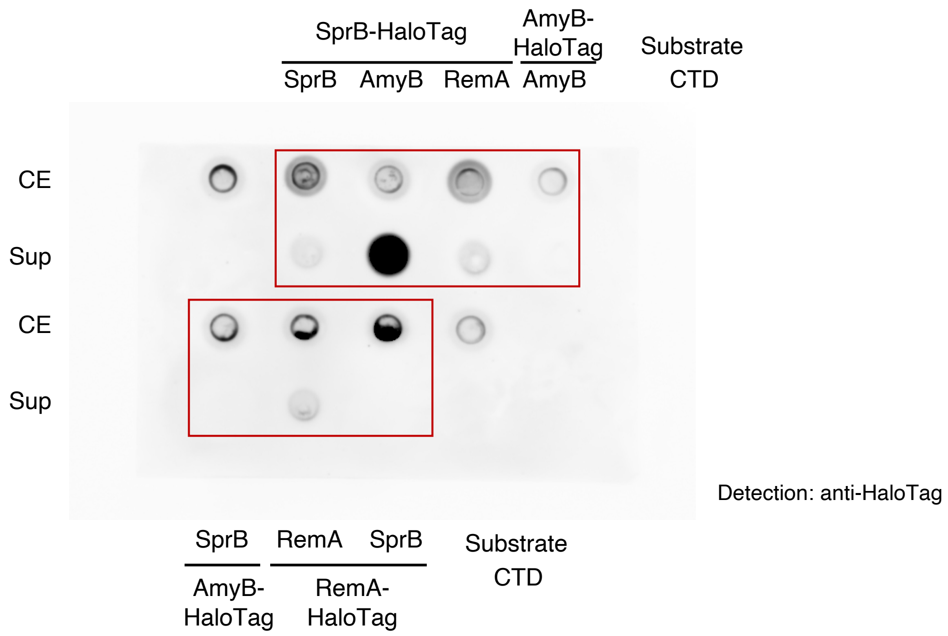

(Supplementary Fig. 5B)

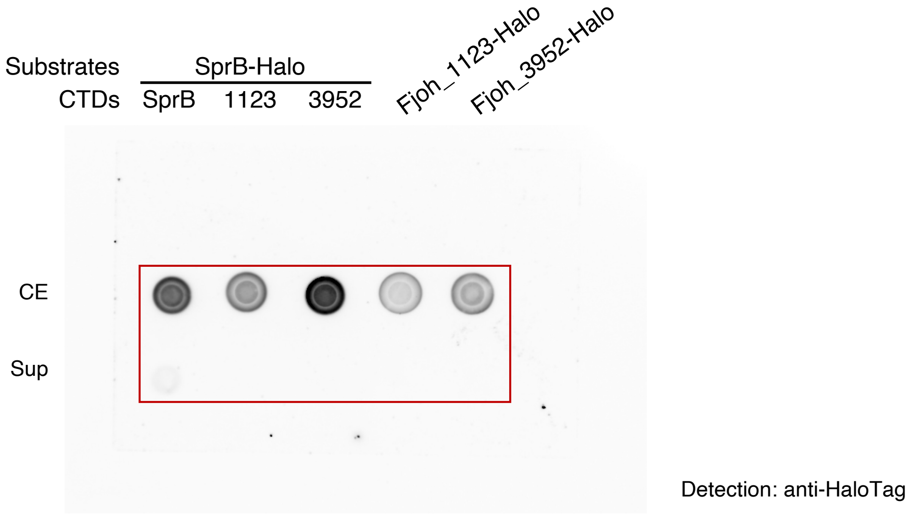

Supplementary Figure 8. Uncropped whole blots of Figures 1B, C, and D, Figure 3B, Figure 4, Figures 5B and C, Figure 6E and Supplementary Figure 5B. Uncropped blots corresponding to each panel in Fig. 1B, 1C, 1D, 3B, 4, 5B, 5C, 6E and Supplementary Fig. 5B are shown. For each blot, the chemiluminescence image (HRP-conjugated antibody detection) and the epi-illumination image (to visualize the Prestained Protein Ladder, Euromedex) are provided. Molecular weight markers (in kDa) are indicated on right (Prestained Protein Ladder, Euromedex). Blots are labeled as shown in the corresponding figure. Red rectangles correspond to the cropped portions of the blots used in the corresponding figure. The antibodies used for each immuno-detection is indicated.

Fusion	Molecular mass
sfGFP-CTD$_{AmyB}$	32.5 kDa
sfGFP-CTD$_{RemA}$	38.3 kDa
sfGFP-CTD$_{SprB}$	51.8 kDa
sfGFP-CTD$_{3952}$	52.2 kDa
sfGFP-CTD$_{1123}$	55 kDa
sfGFP-CTD$_{1720}$	52.4 kDa
sfGFP-CTD$_{4934}$	52.6 kDa
sfGFP	27.1 kDa

Supplementary Table 1. Molecular mass of the sfGFP-CTD fusions used.

Protein	IBAQ (log2)	
	GFP-CTD_{AmyB}	SP-GFP
**AmyB**	24.28	NaN
**PorV**	22.54	15.67
**SprA**	20.88	15.66
**Ppi**	19.90	13.78

Protein	IBAQ (log2)	
	GFP-CTD_{RemA}	SP-GFP
**RemA**	28.71	17.41
**PorV**	24.14	15.18
**SprA**	20.78	14.17
**Ppi**	19.60	NaN

Protein	IBAQ (log2)	
	GFP-CTD_{SprB}	SP-GFP
**SprB**	23.47	17.97
**SprF**	19.25	16.43
**SprA**	19.28	14.17
**Ppi**	17.05	NaN

Protein	IBAQ (log2)	
	GFP-CTD_{3952}	SP-GFP
**Fjoh_3952**	28.47	14.65
**Fjoh_3951**	29.05	15.86
**SprA**	19.01	15.66
**Ppi**	17.98	13.78

Protein	IBAQ (log2)	
	GFP-CTD_{1123}	SP-GFP
**Fjoh_1123**	26.55	13.90
**Fjoh_1677**	17.60	NaN
**SprA**	14.55	15.66

Protein	IBAQ (log2)	
	GFP-CTD_{1720}	SP-GFP
**Fjoh_1720**	25.65	15.65
**Fjoh_1677**	20.56	NaN
**SprA**	12.66	13.21

Protein	IBAQ (log2)	
	GFP-CTD_{4934}	SP-GFP
**Fjoh_4934**	27.45	15.89
**Fjoh_1677**	24.71	13.75
**SprA**	13.42	12.17

Supplementary Table 2. Abundance of identified proteins during co-immunoprecipitation for each sfGFP-CTD fusion relative to the $sfGFP_{peri}$ control based on IBAQ.

SUPPLEMENTARY TABLE 3. Strains with chromosomal constructs used in this study.

STRAINS

Strains	Description	Source or Reference
***Escherichia coli* strains**		
**DH5α**	Strain used for general cloning	Laboratory collection
**HB101**	Strain containing the pRK2013 plasmid for triparental conjugation	Laboratory collection
**S17-1 λ pir**	Strain used for conjugation	Laboratory collection
***Flavobacterium johnsoniae* strains (all derived from the Sm Resistant strain CJ1827)**		
**CJ1827**	rpsL2 Smr "wild-type" F. johnsoniae strain used in construction mutants	Rhodes *et al*., 2011
sprB-halotag-CTD	HaloTag insertion upstream SprB CTD	This study
sprB-halotag-CTD_{AmyB}	CTD of AmyB fused to the SprB-HaloTag fusion	This study
sprB-halotag-CTD_{RemA}	CTD of RemA fused to the SprB-HaloTag fusion	This study
amyB-halotag-CTD	HaloTag insertion upstream AmyB CTD	This study
amyB-halotag-CTD_{SprB}	CTD of SprB fused to the AmyB-HaloTag fusion	This study
remA-halotag-CTD	HaloTag insertion upstream RemA CTD	This study
remA-halotag-CTD_{SprB}	CTD of SprB fused to the RemA-HaloTag fusion	This study
sprB-halotag-CTDFjoh_1123	CTD of Fjoh_1123 fused to the SprB-HaloTag fusion	This study
sprB-halotag-CTDFjoh_3952	CTD of Fjoh_3952 fused to the SprB-HaloTag fusion	This study
Fjoh_1123-halotag-CTD	HaloTag insertion upstream Fjoh_1123 CTD	This study
Fjoh_3952-halotag-CTD	HaloTag insertion upstream Fjoh_3952 CTD	This study
sprF-ALFA	ALFA tag fused to the C-terminus of SprF	This study
Fjoh_3951-ALFA	ALFA tag fused to the C-terminus of Fjho_3951	This study

**pTM218**	Encodes SP_{RemA}-sfGFP-CTD_{Fjoh_392} and Fjoh_3952-ALFA; Amp^R-Tc^R	This study
**pTM220**	Encodes SP_{RemA}-sfGFP-$CTD_{SprB-motA}$ and SprF; Amp^R-Tc^R	This study
**pTM221**	Encodes SP_{RemA}-sfGFP-$CTD_{SprB-motB}$ and SprF; Amp^R-Tc^R	This study
**pTM222**	Encodes SP_{RemA}-sfGFP-$CTD_{SprB-motC}$ and SprF; Amp^R-Tc^R	This study
**pTM223**	Encodes SP_{RemA}-sfGFP-$CTD_{SprB-motD}$ and SprF; Amp^R-Tc^R	This study
**pTM224**	Encodes SP_{RemA}-sfGFP-$CTD_{SprB-motE}$ and SprF; Amp^R-Tc^R	This study
**<u>Suicide plasmids for chromosomal constructs</u>**		
**pRR51-SprB-HaloTag-CTD**	Construct used to generate SprB-HaloTag-CTD fusion; Amp^R-Em^R	This study
**pRR51-SprB-HaloTag-CTD_{AmyB}**	Construct used to generate SprB-HaloTag-CTD_{AmyB} fusion; Amp^R-Em^R	This study
**pRR51-SprB-HaloTag-CTD_{RemA}**	Construct used to generate SprB-HaloTag-CTD_{RemA} fusion; Amp^R-Em^R	This study
**pRR51-AmyB-HaloTag-CTD**	Construct used to generate AmyB-HaloTag-CTD fusion; Amp^R-Em^R	This study
**pRR51-AmyB-HaloTag-CTD_{SprB}**	Construct used to generate AmyB-HaloTag-CTD_{SprB} fusion; Amp^R-Em^R	This study
**pRR51-RemA-HaloTag-CTD**	Construct used to generate RemA-HaloTag-CTD fusion; Amp^R-Em^R	This study
**pRR51-RemA-HaloTag-CTD_{SprB}**	Construct used to generate RemA-HaloTag-CTD_{SprB} fusion; Amp^R-Em^R	This study
**pRR51-SprB-HaloTag-CTD_{Fjoh_1123}**	Construct used to generate SprB-HaloTag-CTD_{Fjoh_1123} fusion; Amp^R-Em^R	This study
**pRR51-SprB-HaloTag-CTD_{Fjoh_3952}**	Construct used to generate SprB-HaloTag-CTD_{Fjoh_3952} fusion; Amp^R-Em^R	This study
**pRR51-Fjoh_1123-HaloTag-CTD**	Construct used to generate Fjoh_1123-HaloTag-CTD fusion; Amp^R-Em^R	This study
**pRR51-Fjoh_3952-HaloTag-CTD**	Construct used to generate Fjoh_3952-HaloTag-CTD fusion; Amp^R-Em^R	This study
**pRR51-sprF-ALFA**	Construct used to generate SprF-ALFA expressed at the native locus; Amp^R-Em^R	This study
**pRR51-Fjoh_3951-ALFA**	Construct used to generate Fjoh_3951-ALFA expressed at the native locus; Amp^R-Em^R	This study

SUPPLEMENTARY TABLE 4. List of all plasmids used in this study.

PLASMIDS

Plasmid	Description and main characteristics	Source or Reference
**pRR51**	Suicide vector containing a wild-type *rpsL* allele, Amp^R-Em^R	Rhodes *et al*. 2011
**Replicative plasmids for overexpression**		
**pSK30**	Encodes SP_{RemA}-sfGFP-CTD_{RemA}; Amp^R-Tc^R	Kulkarni *et al.* 2017
**pSK37**	Encodes SP_{RemA}-sfGFP; Amp^R-Tc^R	Kulkarni *et al.* 2017
**pSK55**	Encodes SP_{RemA}-sfGFP-CTD_{SprB} and SprF; Amp^R-Tc^R	Kulkarni *et al.* 2019
**pSK56**	Encodes SP_{RemA}-sfGFP-CTD_{SprB}; Amp^R-Tc^R	Kulkarni *et al.* 2017
**pSK57**	Encodes SP_{RemA}-sfGFP-CTD_{Fjoh_3952} and Fjoh_3951; Amp^R-Tc^R	Kulkarni *et al.* 2019
**pSK82**	Encodes SP_{RemA}-sfGFP-CTD_{AmyB}; Amp^R-Tc^R	Kulkarni *et al.* 2017
**pMP12**	Encodes SP_{RemA}-sfGFP-CTD_{Fjoh_1123}; Amp^R-Tc^R	This study
**pMP53**	Encodes SP_{RemA}-sfGFP-CTD_{Fjoh_1720}; Amp^R-Tc^R	This study
**pMP54**	Encodes SP_{RemA}-sfGFP-CTD_{Fjoh_4934}; Amp^R-Tc^R	This study
**pMP73**	Encodes SP_{RemA}-sfGFP-$CTD_{SprB-motEFjoh\_3952}$ and SprF; Amp^R-Tc^R	This study
**pMP74**	Encodes SP_{RemA}-sfGFP-$CTD_{SprB-motEFjoh\_3952}$ and Fjoh_3951; Amp^R-Tc^R	This study
**pMP75**	Encodes SP_{RemA}-sfGFP-CTD_{Fjoh_4934} and Fjoh_1677; Amp^R-Tc^R	This study
**pMP76**	Encodes SP_{RemA}-sfGFP-CTD_{Fjoh_1123} and Fjoh_1677; Amp^R-Tc^R	This study
**pMP77**	Encodes SP_{RemA}-sfGFP-CTD_{Fjoh_1720} and Fjoh_1677; Amp^R-Tc^R	This study
**pMP78**	Encodes SP_{RemA}-sfGFP-$CTD_{SprB-motBFjoh\_3952}$ and Fjoh_3951; Amp^R-Tc^R	This study
**pMP79**	Encodes SP_{RemA}-sfGFP-$CTD_{SprB-motBEFjoh\_3952}$ and Fjoh_3951; Amp^R-Tc^R	This study
**pMP80**	Encodes SP_{RemA}-sfGFP-$CTD_{SprB-motBFjoh\_3952}$ and SprF; Amp^R-Tc^R	This study
**pMP81**	Encodes SP_{RemA}-sfGFP-$CTD_{SprB-motBEFjoh\_3952}$ and SprF; Amp^R-Tc^R	This study
**pMP82**	Encodes SP_{RemA}-sfGFP-CTD_{Fjoh_3952} and Fjoh_1646; Amp^R-Tc^R	This study
**pMP83**	Encodes SP_{RemA}-sfGFP-CTD_{Fjoh_3952} and Fjoh_3972; Amp^R-Tc^R	This study
**pMP84**	Encodes SP_{RemA}-sfGFP-CTD_{Fjoh_3952} and SprF; Amp^R-Tc^R	This study
**pMP88**	Encodes SP_{RemA}-sfGFP-CTD_{Fjoh_3971} and Fjoh_3951; Amp^R-Tc^R	This study
**pMP89**	Encodes SP_{RemA}-sfGFP-CTD_{SprB} and Fjoh_3951; Amp^R-Tc^R	This study
**pTM217**	Encodes SP_{RemA}-sfGFP-CTD_{SprB} and SprF-ALFA; Amp^R-Tc^R	This study

SUPPLEMENTARY TABLE 5. Oligonucleotides used in this study.

OLIGONUCLEOTIDES

**Oligonucleotides**	**Sequence 5' to 3'**
**oTM743**	CAGTACCGATTTCGGAACCTGCAGCCATATTAGACTGTGCATTGTT
**oTM744**	GGTTCCGAAATCGGTACTG
**oTM745**	ACCGGAAATCTCCAGAGTAG
**oTM746**	CTACTCTGGAGATTTCCGGTCCTGTGGTGATTACAATTGATCC
**oTM964**	CGGAAAAATTCGGGGGATCCTCTAGAGATGGTACTATCACAGCGAC
**oTM965**	TTGCATGCCTGCAGGTCGACTCTAGACCATGAAACCAGCCATTGGC
**oTM1003**	CCCGAATTTTTCCGCTGCAT
**oTM1004**	GGCGTAATCATGGTCATAGCTG
**oTM1008**	GACG*TCTAGA*TTCGTAAATGATCTGCCAACAG
**oTM1009**	GACG*GCATGC*ATAAATGTTTGAATGCCATCTCCT
**oTM1219**	CGGAAAAATTCGGGGGATCCTCTAGAGTCTGCTCCTGTAGTTTCTC
**oTM1220**	CAGTACCGATTTCGGAACCTGCAGCATCAAATCTCATAATTGTCCA
**oTM1221**	CTACTCTGGAGATTTCCGGTGCTGCACAGCTTACACCTTCAATCGA
**oTM1222**	TTGCATGCCTGCAGGTCGACTCTAGAGTTTCATACGGAAGCAGCAA
**oTM1469**	CGGAAAAATTCGGGGGATCCTCTAGAACAATTTCTGGACCAATGCCAGA
**oTM1470**	CGAATGGAAAGCCAGTACCGATTTCCATATTAGACTGTGCATTGTTAACAC
**oTM1471**	GGCTGTCTACTCTGGAGATTTCCGGTGATCGTTTTGCACTTCGTTACAC
**oTM1472**	TTGCATGCCTGCAGGTCGACTCTAGATGTGCTCTGTGTGCTGTTCCAA
**oTM1473**	CGGAAAAATTCGGGGGATCCTCTAGACTGCCACGCGGCAACTAGTC
**oTM1474**	CGAATGGAAAGCCAGTACCGATTTCTGTAAAAGTTCCCGCTTCTGTT
**oTM1475**	GGCTGTCTACTCTGGAGATTTCCGGTGTGGTGATTACAATTGATCCAAGC
**oTM1477**	TTGCATGCCTGCAGGTCGACTCTAGAGGCGATTACAACATTTACCCAGGAT
**oTM1478**	TAAAAAATCCATTTGCCAAGTAAGCAAAATAATAGCCCCTATGATGTTATCT
**oTM1576**	GGCTGTCTACTCTGGAGATTTCCGGTGAACCAACAACTGTTGGAACAGGA
**oTM1579**	AGATAACATCATAGGGGCTATTATTTTGCGAATCGAACAATAGCGAACAAGC

**oTM1580**	CAAAATAATAGCCCCTATGATGTTATCT
**oTM1585**	CGGAAAAATTCGGGGGATCCTCTAGATATAGATGTTCCTCCAGCTATAGGTT
**oTM1586**	CGAATGGAAAGCCAGTACCGATTTCATCATTTACGAATAAGTCTAAACTTGTTTCAG
**oTM1587**	GGCTGTCTACTCTGGAGATTTCCGGTCTGCCAACAGTAATGATTGAAAAAAC
**oTM1588**	TTGCATGCCTGCAGGTCGACTCTAG*A*AAGCTTTAGCTTTTGACCTTGGAG
**oTM1634**	TGGT*TCTAGA*CCGGTTGTCACCAACGAAAA
**oTM1635**	CTAG*GCATGC*GACGTTTCTGATCGCGATTTTG
**oTM1636**	TGGT*TCTAGA*GATCCGCCAAATTTTGAAAATGAATC
**oTM1637**	CTAG*GCATGC*GGTATAGTTCGGATTCGGACT
**oTM1638**	CGGAAAAATTCGGGGGATCCTCTAGAGAGAAGGTGTATTGATTGCTGC
**oTM1639**	CCGTTTACAGATTTTATAACCATAATATTTTTAATGGTTAGCTAGTTTATATAATTTTTGCTCT
**oTM1640**	AAAAATATTATGGTTATAAAATCTGTAAACGG
**oTM1641**	TTGCATGCCTGCAGGTCGACTCTAGACAGCAGACTGATTATACTTCGG
**oTM1677**	CGGAAAAATTCGGGGGATCCTCTAGAGACGAGGCGATTTAACGACG
**oTM1678**	CGAATGGAAAGCCAGTACCGATTTCTAATTGCCATTGCGCGCTGT
**oTM1679**	TTGCATGCCTGCAGGTCGACTCTAGATGATAATGCTCAATTACATCTTCGC
**oTM1680**	TTATCTGTATAAAGTGAAATGTCCAACAAAC
**oTM1681**	GTTTGTTGGACATTTCACTTTATACAGATAAGCTTGTTCGCTATTGTTCGATTCG
**oTM1705**	TCTAGAACCAGAACCACCACC
**oTM1721**	CGGAAAAATTCGGGGGATCCTCTAGAGGTTAATCCGGTTAACGATGC
**oTM1722**	CGAATGGAAAGCCAGTACCGATTTCGGCGGCTTCGACAAAAATCTTAACTATTGCATCT
**oTM1723**	GGCTGTCTACTCTGGAGATTTCCGGTGTGCCATCGATTACAGTAGTTATGA
**oTM1724**	TTGCATGCCTGCAGGTCGACTCTAGACGCAGGAAGATTTCATGCGAG
**oTM1786**	GTGGTGGTTCTGGTTCTAGAGTGGTGATTACAATTGATCCAAGCA
**oTM1788**	CAAAATAATAGCCCCTATGA
**oTM1791**	TAGTCCAAATCAATAAAATGGCTTAGC
**oTM1822**	GTTGGACATTTCACTTTATACAGATAAAATTACACTTCTAAACCCAAATAAAAAATCCAT
**oTM1908**	GGAGATGGCATTCAAACATTTATCGTACGAAATTAATAAGAGAGCAAAA
**oTM1909**	CGCCAAGCTTGCTAGGCATGCTTAATTAACTGCAGGGCAATTAC
**oTM1988**	ATAAATGTTTGAATGCCATCTCCT
**oTM1989**	GCATGCCTAGCAAGCTTGGCG

**oTM2005**	GCATGCCTAGCAAGCTTGGC
**oTM2007**	GCCAAGCTTGCTAGGCATGCTTCATTTTCATAATTAGAAAAATCTTGGT
**oTM2008**	TTATCTGTATAAAGTGAAATGTCCAACAA
**oTM2009**	ATTTCACTTTATACAGATAATAGTCCAAATCAATAAAATGGCTTA
**oTM2018**	CAAAATCGCGATCAGAAACGTCCGTACGAAATTAATAAGAGAGCAA
**oTM2019**	GACGTTTCTGATCGCGATTTTG
**oTM2020**	GCTCAGCTTATCGTAAGCCGGCTAAGCCATTTTATTGATTTGGA
**oTM2021**	CGGCTTACGATAAGCTGAGC
**oTM2022**	CGCCAAGCTTGCTAGGCATGCTTAAAAGAATCTTGGTGAGGTAAGTTTG
**oTM2026**	AGATAACATCATAGGGGCTATTATTTTGATAAATGTTTGAATGCCATCTCCTTAAT
**oTM2027**	CAAAATAATAGCCCCTATGATGTTATCT
**oTM2028**	AGATAACATCATAGGGGCTATTATTTTGGGCTTAACCTAATGGCTAAGCC
**oTM2029**	AGATAACATCATAGGGGCTATTATTTTGGGTATAGTTCGGATTCGGACTTTAG
**oTM2031**	TCATAGGGGCTATTATTTTGGCTAAGCCATTTTATTGATTTGGA
**oTM2032**	CGCCAAGCTTGCTAGGCATGCGTGCATCATTACAACGAAAACG
**oTM2036**	CGCCAAGCTTGCTAGGCATGCGCCAGAAGTAGGAGTATAGTAATTCTT
**oTM2038**	AATAGGATAACCAAAATAAGAATTGGCTATG
**oTM2041**	TCATAGGGGCTATTATTTTGTTATCTGTATAAAGTGAAATGTCCAACAAAC
**oTM2042**	TAAGCCATTTTATTGATTTGGACTATTATCTGTATAAAGTGAAATGTCCAACAAAC
**oTM2045**	TCATAGGGGCTATTATTTTGTTATTTAATTAG
**oTM2046**	GCTAAGCCATTTTATTGATTTGGACTATTATTT
**oTM2060**	CAAAATAATAGCCCCTATGATGTTATCT
**oTM2501**	ATGCAGCGGAAAAATTCGGGGGTGATGGAGTTAACGATACTT
**oTM2502**	TTATTCTGTTAAACGTCTACGAAGT
**oTM2503**	CTCGTAGACGTTTAACAGAATAATAATTACAATTAAAAACTTACTGTAAATCA
**oTM2504**	CAGCTATGACCATGATTACGCCCTGCTGCAACAGAATCACCTC
**oTM2505**	ATGCAGCGGAAAAATTCGGGCGGAGATAATATGAACGAGAGAT
**oTM2506**	TCGTAGACGTTTAACAGAATAATTATGAAAATGAAAAAAATAGTATATACAC
**oTM2507**	GCTATGACCATGATTACGCCCCGGTTAAAGTTCTTTCGCA

[a] restriction site in italic.

[b] sequence annealing on the target vector underlined

SUPPLEMENTARY TABLE 6. Cloning description of the suicide plasmids generated in this study.

Plasmid name	PCR fragments[a, b]	Primers[c]	Vector
sprB-haloTag-CTD	*sprB*-upCTD	**oTM964** - oTM743	pRR51-XbaI
	haloTag	oTM744 - oTM745	
	CTD-down-*sprB*	oTM746 - **oTM965**	
sprB-haloTag-CTD$_{AmyB}$	*sprB*-upCTD	**oTM1469** - oTM1470	pRR51-XbaI
	haloTag	oTM744 - oTM745	
	CTD_{AmyB}	oTM1576 - oTM1579	
	down-*sprB* for Amy	oTM1580 - **oTM1472**	
sprB-haloTag-CTD$_{RemA}$	*sprB*-upCTD	**oTM1469** - oTM1470	pRR51-XbaI
	haloTag	oTM744 - oTM745	
	CTD_{RemA}	oTM1471 - oTM1475	
	down-*sprB* for RemA	oTM1478 - **oTM1472**	
amyB-haloTag-CTD	*amyB*-upCTD	**oTM1677** - oTM1678	pRR51-XbaI
	HaloTag	oTM744 - oTM745	
	down-*amyB*	oTM1576 - **oTM1679**	
amyB-haloTag-CTD$_{SprB}$	amyB-upCTD	**oTM1677** - oTM1678	pRR51-XbaI
	HaloTag	oTM744 - oTM745	
	CTD_{SprB}	oTM1475 - oTM1680	
	down-*amyB* for SprB	oTM1681 - **oTM1679**	
remA-haloTag-CTD	*remA*-upCTD	**oTM1473** - oTM1474	pRR51-XbaI
	HaloTag	oTM744 - oTM745	
	CTD-down-*remA*	oTM1471 - **oTM1477**	
remA-haloTag-CTD$_{SprB}$	*remA*-upCTD	**oTM1473** - oTM1474	pRR51-XbaI
	HaloTag	oTM744 - oTM745	
	CTD_{SprB}	oTM1475 - oTM1680	
	down-*remA* for SprB	oTM1822 - **oTM1477**	
fjoh_4750-haloTag-CTD	Fjoh_4750-upCTD	**oTM1219** - oTM1220	pRR51-XbaI
	HaloTag	oTM744 - oTM745	
	CTD-down-Fjoh_4750	oTM1221 - **oTM1222**	
fjoh_1123-haloTag-CTD	Fjoh_1123-upCTD	**oTM1585** - oTM1586	pRR51-XbaI
	HaloTag	oTM744 - oTM745	
	CTD-down-Fjoh_1123	oTM1587 - **oTM1588**	
fjoh_3952-haloTag-CTD	Fjoh_3952-upCTD	**oTM1721** - oTM1722	pRR51-XbaI
	HaloTag	oTM744 - oTM745	
	CTD-down-Fjoh_3952	oTM1587 - **oTM1588**	
sprB-haloTag-CTD$_{Fjoh\_1123}$	pRR51-SprB-HaloTag-downSprB	oTM1721 - oTM1722	
	CTD_{Fjoh_1123}	oTM1587 - oTM2026	
sprB-haloTag-CTD$_{Fjoh\_3952}$	pRR51-SprB-HaloTag-downSprB	oTM745 - oTM2027	
	CTD_{Fjoh_3952}	oTM1723 - oTM2028	
sprF-ALFA	pRR51	oTM1003 - oTM1004	
	sprF-up-ALFA	oTM2501 – oTM2502	
	ALFA-down-*sprF*	oTM2503 – oTM2504	
fjoh_3951-ALFA	pRR51	oTM1003 - oTM1004	
	fjoh_3951-up-ALFA	oTM2505 – oTM2502	
	ALFA-down-*fjoh_3951*	oTM2506 – oTM2507	

[a] « up » fragments correspond to the region directly upstream of the CTD, « CTD-down » fragments correspond to the region encoding the CTD and the region directly downstream of the CTD, and « down » fragments correspond to the region directly downstream of the gene.

[b] all fragments except HaloTag and pRR51-SprB-HaloTag-downSprB were PCR amplified from *F. johnsoniae* genomic DNA. HaloTag fragment was amplified from plasmid pSU005 (Banaz et al., 2019). pRR51-SprB-HaloTag-downSprB was amplified from genomic DNA of the SprB-HaloTag-CTD strain.

[c] primers in bold were used to re-amplified the assembled fragment, obtained after Gibson isothermal assembly of the three to four fragments initially obtained (PCR fragment).

Supplementary Methods

Suicide plasmids

The suicide plasmid designed to generate the SprB-HaloTag-CTD fusion was made as follows. A 1.5-kb fragment containing *sprB*, directly upstream of the CTD encoding sequence, was PCR amplified using oligonucleotide primers oTM964 and oTM743 on *F. johnsoniae* genomic DNA matrix. For clarity, this will not be repeated in subsequent sections of the text, but unless otherwise specified, the matrix used for PCR amplifications is the genomic DNA of *Flavobacterium*. A 885-bp fragment encoding HaloTag was PCR amplified using oligonucleotides primers oTM744 and oTM745 on plasmid matrix pSU005[1]. For the next sections, unless otherwise specified, the matrix used for PCR amplification of HaloTag is pSU005[1]. A 1.5-kb fragment containing the CTD and the region directly downstream *sprB* was PCR amplified using oligonucleotide primers oTM746 and oTM965. These fragments were assembled using Gibson isothermal reaction and reamplified using oligonucleotides oTM964 and oTM965. This fragment was inserted into pRR51 digested with *Xba*I using Gibson isothermal reaction.

The suicide plasmid designed to generate the SprB-HaloTag-CTD_{AmyB} fusion was made as follows. A 1.7-kb fragment containing *sprB* was PCR amplified using oligonucleotide primers oTM1469 and oTM1470. A 885-bp fragment encoding HaloTag was PCR amplified using oligonucleotides primers oTM744 and oTM745. A 390-bp fragment containing the CTD of AmyB was PCR amplified using oligonucleotide primers oTM1576 and oTM1579. A 1.7-kb fragment containing the region directly downstream *sprB* was PCR amplified using oligonucleotide primers oTM1580 and oTM1472. These fragments were assembled using Gibson isothermal reaction and reamplified using oligonucleotides oTM1469 and oTM1472. This fragment was inserted into pRR51 digested with *Xba*I using Gibson isothermal reaction.

The suicide plasmid designed to generate the SprB-HaloTag-CTD_{RemA} fusion was made as follows. A 1.7-kb fragment containing *sprB* was PCR amplified using oligonucleotide primers oTM1469 and oTM1470. A 885-bp fragment encoding HaloTag was PCR amplified using oligonucleotides primers oTM744 and oTM745. A 339-bp fragment containing the CTD of RemA was PCR amplified using oligonucleotide primers oTM1471 and oTM1475. A 1.7-kb fragment containing the region directly downstream *sprB* was PCR amplified using oligonucleotide primers oTM1478 and oTM1472. These fragments were assembled using

Gibson isothermal reaction and reamplified using oligonucleotides oTM1469 and oTM1472. This fragment was inserted into pRR51 digested with *Xba*I using Gibson isothermal reaction.

The suicide plasmid designed to generate the AmyB-HaloTag-CTD fusion was made as follows. A 1.7-kb fragment containing *amyB* was PCR amplified using oligonucleotide primers oTM1677 and oTM1678. A 885-bp fragment encoding HaloTag was PCR amplified using oligonucleotides primers oTM744 and oTM745. A 1.7-kb fragment containing the CTD and region directly downstream *amyB* was PCR amplified using oligonucleotide primers oTM1576 and oTM1679. These fragments were assembled using Gibson isothermal reaction and reamplified using oligonucleotides oTM1677 and oTM1679. This fragment was inserted into pRR51 digested with *Xba*I using Gibson isothermal reaction.

The suicide plasmid designed to generate the AmyB-HaloTag-CTD_{SprB} fusion was made as follows. A 1.7-kb fragment containing *amyB* was PCR amplified using oligonucleotide primers oTM1677 and oTM1678. A 885-bp fragment encoding HaloTag was PCR amplified using oligonucleotides primers oTM744 and oTM745. A 657-bp fragment containing the CTD of SprB was PCR amplified using oligonucleotide primers oTM1475 and oTM1680. A 1.4-kb fragment containing the region directly downstream *amyB* was PCR amplified using oligonucleotide primers oTM1681 and oTM1679. These fragments were assembled using Gibson isothermal reaction and reamplified using oligonucleotides oTM1677 and oTM1679. This fragment was inserted into pRR51 digested with *Xba*I using Gibson isothermal reaction.

The suicide plasmid designed to generate the RemA-HaloTag-CTD fusion was made as follows. A 1.7-kb fragment containing *remA* was PCR amplified using oligonucleotide primers oTM1473 and oTM1474. A 885-bp fragment encoding HaloTag was PCR amplified using oligonucleotides primers oTM744 and oTM745. A 2.1-kb fragment containing the CTD and region directly downstream *remA* was PCR amplified using oligonucleotide primers oTM1471 and oTM1477. These fragments were assembled using Gibson isothermal reaction and reamplified using oligonucleotides oTM1473 and oTM1477. This fragment was inserted into pRR51 digested with *Xba*I using Gibson isothermal reaction.

The suicide plasmid designed to generate the RemA-HaloTag-CTD_{SprB} fusion was made as follows. A 1.7-kb fragment containing *remA* was PCR amplified using oligonucleotide primers oTM1473 and oTM1474. A 885-bp fragment encoding HaloTag was PCR amplified using

oligonucleotides primers oTM744 and oTM745. A 657-bp fragment containing the CTD of SprB was PCR amplified using oligonucleotide primers oTM1475 and oTM1680. A 2.1-kb fragment containing the region directly downstream *remA* was PCR amplified using oligonucleotide primers oTM1822 and oTM1477. These fragments were assembled using Gibson isothermal reaction and reamplified using oligonucleotides oTM1473 and oTM1477. This fragment was inserted into pRR51 digested with *Xba*I using Gibson isothermal reaction.

The suicide plasmid designed to generate the Fjoh_4750-HaloTag-CTD fusion was made as follows. A 1.7-kb fragment containing Fjoh_4750 was PCR amplified using oligonucleotide primers oTM1219 and oTM1220. A 885-bp fragment encoding HaloTag was PCR amplified using oligonucleotides primers oTM744 and oTM745. A 1.5-kb fragment containing the region directly downstream Fjoh_4750 was PCR amplified using oligonucleotide primers oTM1221 and oTM1222. These fragments were assembled using Gibson isothermal reaction and reamplified using oligonucleotides oTM1219 and oTM1222. This fragment was inserted into pRR51 digested with *Xba*I using Gibson isothermal reaction.

The suicide plasmid designed to generate the Fjoh_1123-HaloTag-CTD fusion was made as follows. A 1.7-kb fragment containing Fjoh_1123 was PCR amplified using oligonucleotide primers oTM1585 and oTM1586. A 885-bp fragment encoding HaloTag was PCR amplified using oligonucleotides primers oTM744 and oTM745. A 1.6-kb fragment containing the region directly downstream Fjoh_1123 was PCR amplified using oligonucleotide primers oTM1587 and oTM1588. These fragments were assembled using Gibson isothermal reaction and reamplified using oligonucleotides oTM1585 and oTM1588. This fragment was inserted into pRR51 digested with *Xba*I using Gibson isothermal reaction.

The suicide plasmid designed to generate the Fjoh_3952-HaloTag-CTD fusion was made as follows. A 1.6-kb fragment containing Fjoh_3952 was PCR amplified using oligonucleotide primers oTM1721 and oTM1722. A 885-bp fragment encoding HaloTag was PCR amplified using oligonucleotides primers oTM744 and oTM745. A 1.6-kb fragment containing the region directly downstream Fjoh_3952 was PCR amplified using oligonucleotide primers oTM1723 and oTM1724. These fragments were assembled using Gibson isothermal reaction and reamplified using oligonucleotides oTM1721 and oTM1724. This fragment was inserted into pRR51 digested with *Xba*I using Gibson isothermal reaction.

The suicide plasmid designed to generate the SprB-HaloTag-CTD_{Fjoh_1123} fusion was made as follows. pRR51-SprB-HaloTag-CTD was amplified using oligonucleotide primers oTM745 and oTM2027. CTD_{Fjoh_1123} sequence was amplified using oligonucleotide primers oTM1587 and oTM2026. These fragments were assembled using Gibson isothermal reaction.

The suicide plasmid designed to generate the SprB-HaloTag-CTD_{Fjoh_3952} fusion was made as follows. pRR51-SprB-HaloTag-CTD was amplified using oligonucleotide primers oTM745 and oTM2027. CTD_{Fjoh_3952} sequence was amplified using oligonucleotide primers oTM1723 and oTM2028. These fragments were assembled using Gibson isothermal reaction.

The suicide plasmid designed to generate the SprF-ALFA, expressed at the native locus, was made as follows. A linear pRR51 backbone fragment was amplified using oligonucleotide primers oTM1003 and oTM1004 on pRR51 plasmid matrix. A 1.3 kb upstream fragment containing *sprF-ALFA* was PCR amplified using oligonucleotide primers oTM2501 and oTM2502 on plasmid pTM217. A 1.3 kb fragment containing the chromosomal region downstream of *sprF* was PCR amplified using oligonucleotide primers oTM2503 and oTM2504. These fragments were assembled using Gibson isothermal reaction.

The suicide plasmid designed to generate the Fjoh_3951-ALFA, expressed at the native locus, was made as follows. A linear pRR51 backbone fragment was amplified using oligonucleotide primers oTM1003 and oTM1004 on pRR51 plasmid matrix. A 1.3 kb upstream fragment containing *fjoh_3951-ALFA* was PCR amplified using oligonucleotide primers oTM2505 and oTM2502 on plasmid pTM218. A 1.3 kb fragment containing the chromosomal region downstream of *fjoh_3951* was PCR amplified using oligonucleotide primers oTM2506 and oTM2507. These fragments were assembled using Gibson isothermal reaction.

Replicative plasmids

Plasmids expressing SP_{RemA}-sfGFP fused to CTD-containing regions of *F. johnsoniae* Fjoh_1123, Fjoh_1720 and Fjoh_4934 were generated as follows. A 762-bp fragment encoding the CTD of Fjoh_1123 was PCR amplified using oligonucleotide primers oTM1008 and oTM1009. This fragment was digested with *Xba*I and *Sph*I and inserted into plasmid pSK30[2] cut with the same restriction enzymes, generating plasmid pMP12. A 821-bp fragment encoding the CTD of Fjoh_1720 was PCR amplified using oligonucleotide primers oTM1634 and

oTM1635. This fragment was digested with *Xba*I and *Sph*I and inserted into plasmid pSK30 cut with the same restriction enzymes, generating plasmid pMP53. A 736-bp fragment encoding the CTD of Fjoh_4934 was PCR amplified using oligonucleotide primers oTM1636 and oTM1637. This fragment was digested with *Xba*I and *Sph*I and inserted into plasmid pSK30 cut with the same restriction enzymes, generating plasmid pMP54.

Plasmid pMP75, which encodes both SP_{RemA}-sfGFP-CTD_{Fjoh_4934} and Fjoh_1677 was constructed as follows. *fjoh_1677* and pMP54 were amplified using oligonucleotide primers oTM1991 and oTM1909, and oTM1989 and oTM1990, respectively. PCR fragments were assembled using Gibson isothermal reaction.

Plasmid pMP76, which encodes both SP_{RemA}-sfGFP-CTD_{Fjoh_1123} and Fjoh_1677 was constructed as follows. *fjoh_1677* and pMP12 were amplified using oligonucleotide primers oTM1908 and oTM1909, and oTM1989 and oTM1988, respectively. PCR fragments were assembled using Gibson isothermal reaction.

Plasmid pMP77, which encodes both SP_{RemA}-sfGFP-CTD_{Fjoh_1720} and Fjoh_1677 was constructed as follows. *fjoh_1677* and pMP53 were amplified using oligonucleotide primers oTM2018 and oTM1909, and oTM1989 and oTM2019, respectively. PCR fragments were assembled using Gibson isothermal reaction.

Plasmids pMP73 and pMP74, which encodes both SP_{RemA}-sfGFP-$CTD_{SprB\text{-}MotEFjoh\_3952}$ and SprF, and SP_{RemA}-sfGFP-$CTD_{SprB\text{-}MotEFjoh\_3952}$ and Fjoh_3951 were synthetized by Twist Bioscience.

Plasmid pMP78, which encodes both SP_{RemA}-sfGFP-$CTD_{SprB\text{-}MotBFjoh\_3952}$ and Fjoh_3951 was constructed as follows. $CTD_{SprB\text{-}MotBFjoh\_3952}$ synthetized gene and pSK57 were amplified using oligonucleotide primers oTM1786 and oTM2042, and oTM1705 and oTM1791, respectively. PCR fragments were assembled using Gibson isothermal reaction.

Plasmid pMP79, which encodes both SP_{RemA}-sfGFP-$CTD_{SprB\text{-}MotBEFjoh\_3952}$ and Fjoh_3951 was constructed as follows. $CTD_{SprB\text{-}MotBEFjoh\_3952}$ synthetized gene and pSK57 were amplified using oligonucleotide primers oTM1786 and oTM2046, and oTM1705 and oTM1791, respectively. PCR fragments were assembled using Gibson isothermal reaction.

Plasmid pMP80, which encodes both SP$_{\text{RemA}}$-sfGFP-CTD$_{\text{SprB-MotBFjoh_3952}}$ and SprF was constructed as follows. CTD$_{\text{SprB-MotBFjoh_3952}}$ synthetized gene and pSK57 were amplified using oligonucleotide primers oTM1786 and oTM2041, and oTM1705 and oTM2060, respectively. PCR fragments were assembled using Gibson isothermal reaction.

Plasmid pMP81, which encodes both SP$_{\text{RemA}}$-sfGFP-CTD$_{\text{SprB-MotBEFjoh_3952}}$ and SprF was constructed as follows. CTD$_{\text{SprB-MotBEFjoh_3952}}$ synthetized gene and pSK55 were amplified using oligonucleotide primers oTM1786 and oTM2045, and oTM1705 and oTM2060, respectively. PCR fragments were assembled using Gibson isothermal reaction.

Plasmid pMP82, which encodes both SP$_{\text{RemA}}$-sfGFP-CTD$_{\text{Fjoh_3952}}$ and Fjoh_1646 was constructed as follows. *fjoh_1646* and pSK57 were amplified using oligonucleotide primers oTM2021 and oTM2022, and oTM2020 and oTM1989, respectively. PCR fragments were assembled using Gibson isothermal reaction.

Plasmid pMP83, which encodes both SP$_{\text{RemA}}$-sfGFP-CTD$_{\text{Fjoh_3952}}$ and Fjoh_3972 was constructed as follows. *fjoh_3972* and pSK57 were amplified using oligonucleotide primers oTM2038 and oTM2036, and oTM2029 and oTM1989, respectively. PCR fragments were assembled using Gibson isothermal reaction.

Plasmid pMP84, which encodes both SP$_{\text{RemA}}$-sfGFP-CTD$_{\text{Fjoh_3952}}$ and SprF was constructed as follows. *sprF* and pSK57 were amplified using oligonucleotide primers oTM1788 and oTM2032, and oTM2031 and oTM1989, respectively. PCR fragments were assembled using Gibson isothermal reaction.

Plasmid pMP84, which encodes both SP$_{\text{RemA}}$-sfGFP-CTD$_{\text{Fjoh_3952}}$ and SprF was constructed as follows. *sprF* and pSK57 were amplified using oligonucleotide primers oTM1788 and oTM2032, and oTM2031 and oTM1989, respectively. PCR fragments were assembled using Gibson isothermal reaction.

Plasmid pMP89, which encodes both SP$_{\text{RemA}}$-sfGFP-CTD$_{\text{SprB}}$ and Fjoh_3951 was constructed as follows. *fjoh_3951* and pSK55 were amplified using oligonucleotide primers oTM2009 and oTM2007, and oTM2008 and oTM2005, respectively. PCR fragments were assembled using Gibson isothermal reaction.

Plasmids pTM217, pTM218, pTM220, pTM221, pTM222, pTM223 and pTM224 were synthesized by Twist Bioscience.

References

1. Banaz, N., Mäkelä, J. & Uphoff, S. Choosing the right label for single-molecule tracking in live bacteria: side-by-side comparison of photoactivatable fluorescent protein and Halo tag dyes. *J Phys D Appl Phys* **52**, 064002 (2019).
2. Kulkarni, S. S., Zhu, Y., Brendel, C. J. & McBride, M. J. Diverse C-Terminal Sequences Involved in Flavobacterium johnsoniae Protein Secretion. *J Bacteriol* **199**, (2017).
3. Abramson, J. *et al.* Accurate structure prediction of biomolecular interactions with AlphaFold 3. *Nature* **630**, 493–500 (2024).
