# Supplementary material for: Specialized shuttle proteins recognize Type IX secretion signals and target effectors to their final destinations in Flavobacterium johnsoniae
Source: Commun Biol. 2025 Nov 14;8:1566. doi: 10.1038/s42003-025-08926-8 (PMC12618676; doi:10.1038/s42003-025-08926-8)
Supplement: Supplementary file 2 — Description of Additional Supplementary Materials [file 42003_2025_8926_MOESM2_ESM.pdf]

Description of Additional Supplementary Files

File name: Supplementary Data

Description: Numerical data used to generate the graphs in Figure 2

File name: Supplementary Movie 1

Description: Time-lapse fluorescence microscopy of SprB-HaloTag-CTD dynamics at the cell surface.

File name: Supplementary Movie 2

Description: Time-lapse fluorescence microscopy of RemA-HaloTag-CTD dynamics, showing immobile foci.

File name: Supplementary Movie 3

Description: Time-lapse fluorescence microscopy of RemA-HaloTag-CTD dynamics, showing highly mobile foci.

File name: Supplementary Movie 4

Description: Time-lapse fluorescence microscopy of AmyB-HaloTag-CTDSprB chimera, showing helical surface movement

File name: Supplementary Movie 5

Description: Time-lapse fluorescence microscopy of RemA-HaloTag-CTDSprB chimera, showing helical surface movement

File name: Supplementary Movie 6

Description: Time-lapse fluorescence microscopy of SprB-HaloTag-CTDRemA chimera, showing static anchoring.

File name: Supplementary Movie 7

Description: Time-lapse fluorescence microscopy of Fjoh_4750-HaloTag-CTD, showing helical surface movement.

File name: Supplementary Movie 8

Description: Time-lapse fluorescence microscopy of SprB-HaloTag-CTDFjoh_1123 chimera, showing subpopulations with static or helical behaviour

File name: Supplementary Movie 9

Description: Time-lapse fluorescence microscopy of Fjoh_3952-HaloTag-CTD, showing static surface anchoring.
